# Chronic Kidney Disease-Associated Itch (CKD-aI) in Children—A Narrative Review

**DOI:** 10.3390/toxins13070450

**Published:** 2021-06-29

**Authors:** Radomir Reszke, Katarzyna Kiliś-Pstrusińska, Jacek C. Szepietowski

**Affiliations:** 1 Department of Dermatology, Venereology and Allergology, Wrocław Medical University, 1 Chałubińskiego Street, 50-368 Wrocław, Poland; radomir.reszke@umed.wroc.pl; 2 Department of Paediatric Nephrology, Wrocław Medical University, 213 Borowska Street, 50-556 Wrocław, Poland

**Keywords:** chronic kidney disease, uraemic toxins, children, itch

## Abstract

Chronic kidney disease (CKD) is a condition of widespread epidemiology and serious consequences affecting all organs of the organism and associated with significant mortality. The knowledge on CKD is rapidly evolving, especially concerning adults. Recently, more data is also appearing regarding CKD in children. Chronic itch (CI) is a common symptom appearing due to various underlying dermatological and systemic conditions. CI may also appear in association with CKD and is termed chronic kidney disease-associated itch (CKD-aI). CKD-aI is relatively well-described in the literature concerning adults, yet it also affects children. Unfortunately, the data on paediatric CKD-aI is particularly scarce. This narrative review aims to describe various aspects of CKD-aI with an emphasis on children, based on the available data in this population and the data extrapolated from adults. Its pathogenesis is described in details, focusing on the growing role of uraemic toxins (UTs), as well as immune dysfunction, altered opioid transmission, infectious agents, xerosis, neuropathy and dialysis-associated aspects. Moreover, epidemiological and clinical aspects are reviewed based on the few data on CKD-aI in children, whereas treatment recommendations are proposed as well, based on the literature on CKD-aI in adults and own experience in managing CI in children.

## 1. Introduction

Itch (also referred to as pruritus) is defined as an unpleasant sensation leading to a desire to scratch. Based on its duration itch is classified as acute or chronic (CI), with the latter being present for more than 6 weeks in an affected individual [1]. CI may manifest in a continuous or intermittent manner, yet its long duration clearly implies an underlying disease-related context. Unsurprisingly, various conditions managed by different medical specialists are prone to be associated with CI. According to the classification proposed by the International Forum for the Study of Itch (IFSI), the aetiological causes of CI may be divided into cutaneous (I), systemic (II), neurologic (III), psychogenic (IV), mixed (V) and other (VI) [2]. Regardless of the common cutaneous diseases mostly managed by dermatologists which frequently present with CI (e.g., atopic dermatitis [AD], urticaria, psoriasis), there is a plethora of other associated chronic conditions of systemic nature, including hepatic, haematologic, oncologic, rheumatologic or renal. Regarding the latter group, a particular emphasis should be given to the spectrum of chronic kidney disease (CKD) which is defined as kidney damage or glomerular filtration rate (GFR) lower than 60 mL/min/1.73 m^2^ for a period of at least 3 months [3]. According to the GFR, five stages of CKD are mentioned, with the fifth stage (GFR < 15 mL/min/1.73 m^2^) also termed uraemia or end-stage renal disease (ESRD). The impact of CKD is vast in all regions of the world. According to Hill et al. [4], the global prevalence of CKD (all stages) was estimated as 13.4%, stages 3–5 affected 10.6% of population, whereas stage 5–0.1%. In 2017 there were 697.5 million new cases of CKD recorded, with 1.2 million of deaths associated with CKD [5]. It is obvious that with such overwhelming epidemiological figures, the everyday clinical practice shall reveal marked heterogeneity in patients’ characteristics, possibly with varying causes of the disease, clinical course, expected consequences and preferable clinical management. This remains valid especially in the context of age groups. Population aging and common crucial risk factors for CKD development, such presence of diabetes and hypertension, have all contributed to the growing magnitude of CKD over the last decades, especially in the elderly [5]. Unfortunately, the global CKD burden also stems from its presence in paediatric population. The prevalence of CKD varies between 15–74.7 per million children in different geographic regions [6]. In 2008, the median reported incidence of renal replacement therapy reached 9 per million children worldwide, with values ranging between 4 to 18 per million in different countries [7]. The aforementioned heterogeneity between different age groups is also expressed via the aetiological factors contributing to the development of CKD. While adults mostly develop CKD due to diabetic nephropathy, hypertension and autosomal dominant polycystic kidney disease (ADPKD), the leading causes in children encompass congenital anomalies of the kidney and urinary tract (CAKUT), hereditary nephropathies and glomerulonephritis [8]. The major CKD complications in children are cardiovascular disease (CVD) presenting with hypertension and/or dyslipidaemia, impaired cognitive development, anaemia, poor or slowed growth, decreased appetite or even death [9]. ESRD is associated with 30 times higher likelihood of mortality than in healthy children [10]. Moreover, young patients with CKD have significantly reduced health-related quality of life (HRQoL) in its various domains [11,12,13]; this also concerns their caregivers [13].

CI is also a frequent problem in the general population. According to Ständer et al. [14], the point-prevalence of CI in adults was 16.8%, whereas Matterne et al. [15] assessed its lifetime prevalence as 25.5%. The burden of CI may manifest with detrimental effect on an individual through decreased mood, reduced concentration, worse sleep quality, suicidal thoughts, difficult socioeconomic situation, feelings of stigmatization or impaired sexual life, to mention just a few [16,17,18,19,20,21]. CKD-associated itch (CKD-aI) is a well-described entity in adults with CKD. Based on recent data, it affects 42–44% of adults undergoing haemodialysis (HD) [22,23], while in patients undergoing peritoneal dialysis (PD) the percentages exceed 60% [24,25]. Patients who underwent successful renal transplantation (RTx) still complain of CI in up to 32% cases, albeit the percentages are lower than during their previous HD sessions [26,27,28]. However, CKD-aI may also bother CKD patients not yet on dialysis, including those with earlier stages of the disease [23,29,30].

Analogously to the CKD population, studies focusing on CI in children are published in the literature less frequently than those referring to the adult population. Notably, CI in paediatric population occurring due to systemic causes has been relatively rarely investigated in the literature [31,32,33,34,35,36,37,38], while publications specifically mentioning CKD-aI appear even more exceptionally [39,40,41,42,43,44]. In this narrative review we attempted to gather the data on CKD-aI in the context of paediatric population and demonstrate the areas which require further evaluation in the future. Due to the scarcity of available papers referring to paediatric population suffering from CKD-aI, a significant proportion of considerations refers to adult CKD-aI and/or paediatric CKD patients, as reported in the literature.

We performed an extensive search of the English literature in PubMed© and Scopus© databases using terms “itch”, “pruritus”, “uraemic”, “kidney”, “renal”, “dialysis”, “children”, and “paediatric”, in different combinations. We also included the data from other relevant online sources on CKD [9,45] which we were aware of. The search was executed on 30 April 2021.

## 2. Pathogenesis of CKD-aI

**Figure 1:**
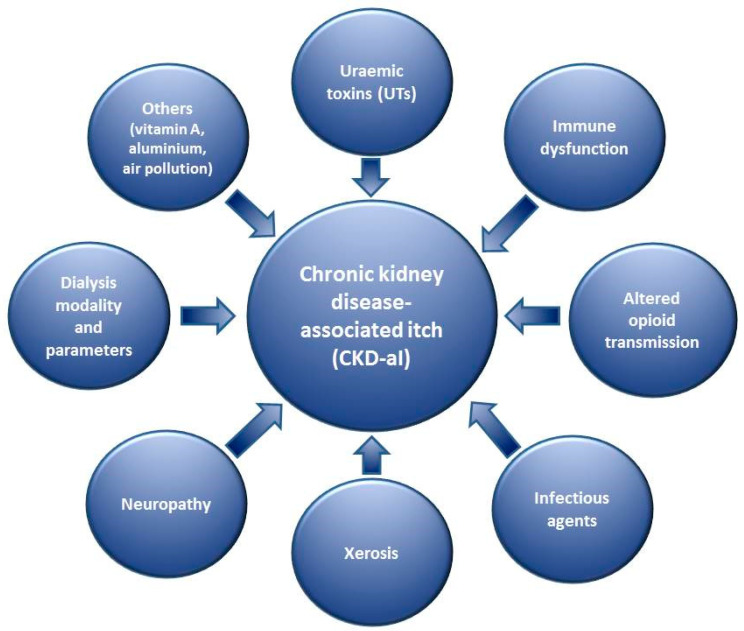
Major groups of pathogenetic factors contributing to the development of CKD-aI.

CKD-aI is a classic example of multifactorial pathogenesis of CI. Table 1/Figure 1 lists the most important factors/groups of factors relevant in CKD-aI pathogenesis. It is important to note that the complicated network of reciprocal associations results in certain overlapping of the pathogenetic mechanisms. Therefore, the separate considerations on subsequent factors are provided in this manner for educational purposes.

### 2.1. Uraemic Toxins (UTs)

Although the term “uraemia” used to be synonymous with all the signs and symptoms of advanced kidney failure, more currently it can be described as an illness accompanying kidney failure that cannot be explained by derangements in extracellular volume, inorganic ion concentrations, or the lack of known renal synthetic products (as reviewed by Meyer and Hostetter) [46]. Uraemic toxins (UTs) constitute a heterogenous group of substances accumulated in uraemia which interact negatively with biologic functions of the organism [47]. Three major groups of substances can be distinguished in the UTs spectrum: free water-soluble low molecular weight molecules (LMWM) (<0.5 kDa), middle molecules (0.5–60 kDa) and protein-bound UTs (PBUT) (Table 2). As of April 2021, the European Uremic Toxins (EUTox) Database [45] lists 130 substances based on 442 source publications and includes their serum concentrations in uraemia in comparison to healthy individuals. It must be noted that such basic small solutes as urea and creatinine are excluded from this classification and it is controversial whether they should be regarded as toxic *per se* [47,48].

It is crucial to acknowledge that the vast majority of data on UT refers to adult population with CKD. As emphasized by Belgian experts in the field [49], publications strictly focusing on paediatric CKD patients are lacking, while the available knowledge regarding the adults may not be fully translated to younger individuals. Therein, the authors mentioned several relevant disparities between paediatric and adult population, mainly larger body water volume and lower circulating proteins, different dietary needs and intake, the ongoing and unfinished processes of maturation and growth, distinct aetiology of CKD, and relatively longer survival when compared to adults with ESRD. Fortunately, as of 2021, new valuable contributions on paediatric CKD and UTs in particular have already been published [50,51,52,53,54,55,56].

#### 2.1.1. Free Water-Soluble Low Molecular Weight Molecules (LMWM)

Low molecular weight molecules (LMWM) are water soluble substances which do not bind to proteins [57]. In comparison to MM and PBUT, they are easily cleared through dialysis membranes. Therefore, patients with ESRD undergoing HD or PD are less likely to accumulate LMWM. Nevertheless, at least one compound in this group was linked to the development of CKD-aI, although any potential direct causal relationship is debatable. Uric acid (UA) is an organic substance which arises as an oxidation end-product of purine metabolism in humans and higher primates. UA is mainly excreted via urine (70%), while gastroenteric elimination accounts for the remaining fraction [58]. Increased UA levels contributed to the risk of CKD disease progression and mortality in adults [59,60]. Similarly, hyperuricaemia was associated with hypertension and CKD progression in children [61]. In a study involving 320 adult patients with CKD (stages 1–5) it was noted that elevated UA levels favoured the development of pruritus in the course of ESRD (*p* = 0.002) [62]. However, Solak et al. [30] evaluated 402 patients with predialysis CKD (stages 2–5) and observed no correlation between UA concentration and the occurrence of pruritus (*p* = 0.133). Moreover, no relation between UA concentration and pruritus was determined in ESRD patients undergoing HD (*n* = 83) [63].

#### 2.1.2. Medium Molecules (MM)

The majority of MM are peptides and proteins which weigh between 0.5 and 60 kDa, with the latter value also constituting the threshold for the glomerular basement membrane filtration [64]. If the dialyser pore is not big enough, the removal of these substances is difficult to obtain during dialysis. Interestingly, with regard to MM and LMWM removal, haemodiafiltration (HDF) proved more effective than HD [47]. According to Chmielewski et al. [64], the spectrum of MM encompasses adipokines, advanced glycation end products (AGEs), appetite regulators, natriuretic peptides, *β*_2_-microglobulin (*β*_2_-M), complement factor D, cystatin C, endothelin-1, free immunoglobulin light chains, interleukins (IL), parathyroid hormone (PTH), fibroblast growth factor-23 (FGF-23), pentraxin-3, prolactin and retinol-binding protein (RBP).

##### β_2_-Microglobulin (β_2_-M)

*β*_2_-M is a 11.8 kDa polypeptide forming *β* chain of the human leukocyte antigen (HLA) class I molecule, thereby participating in antigen presentation to CD8 lymphocytes [65,66]. Its retention in the course of advanced stages of CKD was associated with amyloidosis, malnutrition-inflammation and atherosclerosis syndrome, cardiovascular disease and mortality [67,68,69,70,71,72]. As reviewed by Argyropoulos et al. [73], despite the conflicting data on the usefulness of *β*_2_-M in determining GFR in children, the fractional excretion of *β*_2_-M might still play a role in the diagnosis of tubulo-interstitial diseases in this population. Several studies focusing on CKD-aI in adults assessed its relationship with *β*_2_-M concentration. Narita et al. [74] evaluated 1773 patients on maintenance HD suffering from CDK-aI, among whom 473 reported severe pruritus (at least 7 points according to the Visual Analogue Scale [VAS]). Higher *β*_2_-M concentrations were associated with increased risk of severe pruritus (*p* = 0.0006 compared to groups with mild or moderate pruritus). Moreover, the occurrence of severe pruritus was associated with increased mortality, whereas low *β*_2_-M concentrations seemed to play protective role. The authors hypothesized that increased *β*_2_-M concentration might stimulate production or accumulation of IL-2 or TNF-*α*, with subsequent activation of CD4 lymphocytes, thereby increasing the risk of pruritus. In a study by Chen et al. [75] (*n* = 116), high-permeability HD proved more effective than conventional HD in reducing CKD-aI intensity, as well as resulting in lower *β*_2_-M concentrations. Conversely, in a study by Melo et al. [76] no differences were noted in the concentrations of *β*_2_-M when comparing 101 HD patients with and without pruritus. Despite the fact that skin deposits of *β*_2_-M have been found in individuals undergoing dialysis [77], there is no definite proof that this could directly stimulate pruritus. Nevertheless, a study on mice exhibited dose-dependent scratching behaviour following intradermal injection of *β*_2_-M [78].

##### Parathyroid Hormone (PTH)

PTH is the major product of chief cells in parathyroid glands, with its initial form containing 115 amino acids and termed preproPTH. The active form of the hormone contains 84 amino acids (PTH_(1–84),_ also termed intact PTH [iPTH]) and exerts its effects through specific G-coupled receptors (PTH1R) [79]. PTH is a crucial factor regulating calcium and phosphate homeostasis in the organism, influencing bones, kidneys and gastrointestinal tract [80]. PTH stimulates Ca^2+^ release to the extracellular fluid, influences osteoblasts and osteoclasts in the bones, promotes renal reabsorption of calcium and inhibits reabsorption of phosphate. It also stimulates the synthesis of 1*α*-hydroxylase, which is an enzyme necessary in the synthesis of the biologically active form of vitamin D_3_. In the setting of CKD, hyperphosphataemia, hypocalcaemia and decreased concentration of vitamin D_3_ result in chronic hyperstimulation of parathyroid glands. This perpetuates their increased growth (initially diffuse, then glandular), PTH secretion and release, thereby developing secondary hyperparathyroidism [81]. PTH and FGF-23 are relevant compounds involved in the pathogenesis of CKD mineral and bone disorder (CKD-MBD) in children, influencing bone turnover, mineralization, volume, linear growth, structure and strength, as well as contributing to vascular calcification [82]. Certain data suggest that PTH is another UT possibly contributing to the development of CKD-aI. In 1968, Massry et al. [83] reported on 7 patients with the coexistence of ESRD, secondary hyperparathyroidism and severe intractable pruritus who underwent subtotal parathyroidectomy. Within 48 h of the procedure, pruritus completely disappeared in 5 patients, with the remaining 2 patients experiencing its disappearance within a week. Similar outcomes were reported by Chou et al. [84] (*n* = 37), although the authors found no correlation between iPTH concentration and pruritus, with its postoperative severity positively correlating with calcium phosphorus product (Ca × P). Other studies brought conflicting results, with some demonstrating possible correlation between serum iPTH concentration and pruritus [74,85,86], while others refuted this hypothesis [30,63,87,88,89]. Notably, a study by Senturk et al. [42] conducted among 27 children on PD demonstrated higher serum PTH concentrations among pruritic subjects (*p* = 0.03), similarly to phosphorus, CRP levels and Ca × P (*p* = 0.027; 0.026; *p* = 0.031; respectively). These factors were also independently associated with pruritus in a stepwise logistic regression model. The exact pathogenetic mechanisms linking PTH and CKD-aI are unknown; interestingly, Ståhle-Bäckdahl et al. [90] injected PTH intradermally in healthy controls and patients on HD, with no pruritic response in both groups. Moreover, histochemical analysis of skin biopsies taken from patients on HD was consistently negative for PTH. Possibly, the association of PTH with CKD-aI might be expressed indirectly through the influence on calcium homeostasis. Consequently, several studies revealed that increased levels of serum calcium favoured the occurrence of severe CKD-aI [63,74,89], whereas Momose et al. [91] observed increased calcium concentration in the deepest layers of the epidermis of patients with CKD-aI. This may predispose to degranulation and release of various pruritogenic mediators from mastocytes (MCs) and other cells present in the skin.

##### Interleukin-6 (IL-6)

IL-6 is a glycosylated protein weighing 21–28 kDa and consisting of 4 long *α*-helices [92], similarly to other cytokines from the family (e.g., IL-11, IL-27, leukaemia inhibitory factor, oncostatin M). These cytokines share a common pathway of signaling through receptor unit glycoprotein gp130 [93]. Classic IL-6 signaling occurs when the cytokine interacts with a specific membrane bound receptor (IL-6R) which is present on hepatocytes, megakaryocytes and leukocytes, followed by formation of IL-6R complex with gp130 homodimer [94]. Although many cells do not have membrane bound IL-6R, they may still be involved in the so-called trans-signaling process which occurs due to interaction between IL-6, soluble IL-6R (sIL-6R) and gp130, as the latter is ubiquitous. In short, IL-6 has been attributed to a variety of biological effects, mainly acute phase inflammatory responses, stimulation of lymphocytes, as well as regulation of glucose metabolism and hypothalamic-pituitary-adrenal (HPA) axis [95]. There is a wide range of publications linking IL-6 and disease-related pathogenesis, for example regarding CKD and ESRD. Higher levels of proinflammatory cytokines, including IL-6, were associated with worse renal function during CKD [96] and its rapid progression [97]. Moreover, IL-6 levels were proven factor predicting mortality in patients with ESRD who initiated dialysis [98,99]. Following RTx, patients with ESRD experienced swift decline of serum IL-6 concentration [100]. In children suffering from CKD, the development of anaemia was inversely correlated with IL-6 concentration [101]. Additionally, IL-6 predicted acute malnutrition and growth impairment among children and adolescents with CKD [102]. Certain studies revealed a possible link between IL-6 and pruritus occurrence due to chronic sulphur mustard exposure [103] and in the course of prurigo nodularis (PN) [104]. Kimmel et al. [105] reported a correlation between serum IL-6 concentration and the presence of pruritus among 171 individuals undergoing HD (*p* = 0.019), although there was no correlation between IL-6 and pruritus severity. Similar results were obtained by German investigators (*n* = 39 ESRD patients on HD) who also observed associations between IL-6 and impaired mental well-being [106]. It must be noted that certain data in the literature link IL-6 and other inflammatory cytokines to depression, including patients on HD [107,108]. Concurrently, the presence depression in patients on HD is a predictor of future CKD-aI occurrence [109], whereas the coexistence of depressive symptoms in paediatric CKD population does occur in practice [110,111].

#### 2.1.3. Protein-Bound Uraemic Toxins (PBUT)

In the last decade, PBUT have finally started to gain more scientific attention as substances interfering with essential biochemical functions of the individuals affected with advanced stages of CKD [112]. PBUT are chemicals which circulate in blood in a certain equilibrium between free and protein-bound form [113]. While a significant fraction of a solute is protein-bound, the remainder can be removed both by glomerular filtration and tubular secretion [114]. Additionally, if a potentially toxic solute is bound to proteins, its active free form has lower concentration in the circulation and thus the detrimental biological effect is less pronounced. However, in the setting of ESRD, significant protein binding also leads to a major decrease of solute clearance through dialysis [113]. As reviewed in details by Rysz et al. [115], it is the intestinal microbiome which is crucial in the generation of various UT. Essentially, CKD is associated with upregulation of bacteria possessing certain enzymes (such as urease, uricase, tryptophanase), resulting in the generation of UT, whereas concurrent downregulation of bacteria with beneficial properties (such as those producing short chain fatty acids) plays a role as well. Gryp et al. [116] demonstrated lower abundance of *Streptococcus* spp. and increased abundance of *E. coli* when comparing CKD patients to healthy controls. Among the whole spectrum of PBUT, there are compounds which have been recently explored to a larger extent in the context of CKD and its various complications. Indoxyl sulfate (IS) and *p*-cresyl sulfate (pCS) are one of the first PBUT representatives to be identified and are commonly mentioned together in the CKD literature [114]. It must be noted that their chemical structures differ, as they belong to indoles and phenols, respectively [47]. Nevertheless, regarding the abundant similarities, the origins of both substances stem from intestinal microbiota, their precursors are conjugated with sulfates, and they are produced in relatively large quantities. Lin et al. [117] reported a linear correlation between the concentration of both compounds during CKD. Moreover, both IS and pCS are bound noncovalently to serum albumin, with the same binding sites [113,114]. In paediatric patients suffering from CKD, the concentrations of IS and pCS poorly correlated with GFR estimated by modified Schwartz formula [51]. Lastly, their contribution to the progression of CKD and its wide range of severe complications is significant (including, among others, CKD-aI), as reviewed in subsequent paragraphs.

##### Indoxyl Sulfate (IS)

IS has a molecular weight of 213 g/mol, with a large fraction bound to albumins (90%) [118]. In short, the generation of IS is initiated when dietary tryptophan reaches colon. Next, resident microbes transform this amino acid into indole, which is then absorbed into the bloodstream. Indole is conjugated with sulfates in liver, forming IS. It was determined that patients with advanced hepatic cirrhosis coexisting with CKD exhibited relatively lower concentrations of IS and pCS [119]. The elimination of IS takes place in proximal tubules of the kidneys which are rich in organic anion transporters (OAT1, OAT3) [120]. The latter are multispecific protein transporters for various toxins, nutrients and drugs. Finally, IS reaches the tubular lumen through apical membrane transporters (multidrug resistance protein 4 [MRP4] and breast cancer resistance protein [BCRP]), and a free molecule is secreted to urine [118]. In a study on children with CKD, IS was one of the PBUT accumulating already in early stages of the disease (CKD stages 1 and 2; median concentrations 1.9 higher than in healthy children; *p* < 0.05) [50]. IS has a wide range of biological effects in various cells and organs and its nephrotoxic effects arise due to several mechanisms. IS promotes glomerular fibrosis [121] through the activation of transforming growth factor-*β*1 (TGF-*β*1), tissue inhibitor of metalloproteinase-1 (TIMP-1) and pro-*α*-1(I)-collagen [122]. Stimulation of p53 protein contributes to senescence and dysfunction of proximal tubular cells, possibly through the activation of *β*-galactosidase and *α*-smooth muscle actin (*α*-SMA) [123], whereas induction of cyclooxygenase-2 (COX-2) by IS results in proliferation of mesangial cells [124]. IS stimulates oxidative stress [125,126,127,128], whereby contributing to endothelial senescence [129]. Several studies focused on proinflammatory properties of IS [130,131] and its influence on the function of macrophages [132,133,134], with the latter aspect linking IS to the development of atherosclerosis. Clinical studies in adult people constituted IS as an important compound related to the progression of CKD, associated CVD, infections, glucose intolerance and mortality [117,135,136,137,138,139,140]. Similarly, recent studies in paediatric patients with CKD reported that serum IS concentrations could be associated with lower residual urine volume, faster progression of CKD, coexistence with CVD and increased mortality [52,54,55].

##### *p*-Cresyl Sulfate (pCS)

As for pCS, this compound weighs approximately 188 g/mol and binds to serum albumin in 90–98% [114]. Beginning with tyrosine, this amino acid is transformed into *p*-cresol by microbiota present in colon. Next, aryl sulfotransferases present in the gut mucosa and liver transform *p*-cresol into pCS [141]. A fraction of *p*-cresol is also subjected to glucuronide conjugation and *p*-cresyl glucuronide (pCG) is created. Despite the predilection of *p*-cresol transformed into pCS, the homeostasis in patients with advanced CKD is relatively shifted from pCS to pCG [142]. Similarly to IS, the urinary excretion of pCS is based mostly on tubular secretion through OAT1, OAT3, BCRP and MRP4; with a possible role of organic anion transporting polypeptide 4C1 (OATP4C1) [143]. According to a study by Poesen et al. [143], estimated GFR (eGFR) was a good predictor of pCS and IS clearance during CKD. However, the clearance of pCS was approximately three times lower than for IS, implying different tubular transporter affinities and/or additional transporter systems involved in the process. It was also reported that pCS inhibited MRP4 and BCRP (40% and 25%, respectively), while pCG reduced the activity of MRP4 by 70% [144]. Consequently, this could hamper the transport of other substrates which would exert toxic properties upon their accumulation and additionally perpetuate the progression of CKD. The toxicity of pCS also stems from its ability to enhance NADPH oxidase, whereby stimulating generation of reactive oxygen species and exerting cytotoxic effects on renal tubular cells. Moreover, pCS augmented production of TGF-β1, TIMP-1 and pro-α1(I)-collagen, which are inflammatory cytokines involved in renal fibrosis [145]. Azevedo et al. [146] reported that pCS induced oxidative burst and phagocytosis in macrophages, whereas there was no change in HLA-DR and CD86 expression. Despite the activation of macrophages, pCS could therefore impede antigen processing and diminish adaptive immune response. Studies on mice revealed increased risk of insulin resistance and detrimental effects on osteoblasts after pCS administration [147,148]. Rossi et al. [149] determined the correlation between pCS (free and total) and IL-6 concentrations in patients with CKD (stages 3 and 4). The association between increased pCS concentration and arterial stiffness (assessed by pulse wave velocity) was also noted [149,150]. In patients with CKD (stages 3–5, not yet on dialysis) elevated pCS concentrations were associated with cardiovascular events and dialysis during a 3-year follow-up [151]. Higher concentrations of free pCS were also associated with mortality in several studies, especially in patients on HD [152,153,154]. Unlike IS, pCS concentrations in children with CKD did not correlate with CVD or CKD progression [54,55]. Additionally, pCS was elevated only in later stages of paediatric CKD when compared to healthy controls [50].

##### PBUT and Their Role in Eliciting CKD-aI

To date there is a scarcity of data linking PBUT and CKD-aI. Nonetheless, in view of recent publications reviewed below it seems that this topic should be explored in near future, especially given the developing prospects on diminishing PBUT toxicity in CKD. In 2016, Wang et al. [62] published their report on 320 CKD patients (mostly stages 2 and 3), among whom 112 (35%) reported pruritus. The latter group had significantly higher total serum concentrations of IS (*p* = 0.008) and pCS (*p* < 0.0001). Although the association between serum IS levels and pruritus disappeared after adjustments for glutamic pyruvic transaminase (GPT) and UA concentrations, pCS maintained its association with pruritus through further adjustments with anthropometric variables, fasting glucose, total cholesterol, GPT, UA, albumin, WBC count, and hs-CRP. The severity of pruritus was also associated with the total pCS concentration (*p* = 0.0002). The authors discussed the possibility of a proinflammatory effect induced by pCS which could account for their findings. However, other investigators did not observe correlations between the concentrations of PBUT (IS, pCS, phenyl sulfate and hippuric acid) and severity of pruritus in 135 HD patients [155]. Recently, Kim et al. [156] have performed a complex study providing further insights on PBUT and their association with CI. The cultures of normal human epidermal keratinocytes (NHEK) were exposed to IS, pCS or sera obtained from CKD-aI on HD. Each of these procedures resulted in significant increase of protease-activated receptor-2 (PAR-2) mRNA and protein expression. PAR-2 expression was also more pronounced when comparing skin samples of patients with CKD-aI to healthy controls, as well as mice with CKD to healthy ones. The findings of this study are important due to several reasons. Receptors belonging to PAR family participate in non-histaminergic pruritus, especially in the course of AD [157,158,159]. Previously, higher protease activity has also been documented in ESRD patients suffering from pruritus (*n* = 12), in contrast to non-pruritic ESRD patients (*n* = 4) and healthy controls (*n* = 6). The expression of PAR-2 has also correlated with pruritus severity [160]. It remains unknown whether this particular pathway could, and to what extent, contribute to the development or exacerbation of CKD-aI in children.

### 2.2. Immune Dysfunction

As reviewed by Cohen [161], the state of uraemia is irreversibly connected to immune dysfunction. Disturbed immune response contributes to the development of CVD and infections, subsequently leading to mortality. Physiologically, kidneys are responsible for clearing bacterial toxins and various endogenous proteins, e.g., cytokines, whereas resident dendritic cells (DCs) participate in maintaining peripheral tolerance [162]. In the setting of CKD these functions become altered; on the other hand, kidneys are also more susceptible to immune-mediated damage themselves. There are multiple immune mechanisms which occur during the course of CKD and concurrently predispose to pruritus. As reported by Kimmel et al. [105], the Th_1_/Th_2_ lymphocytes proportion (assessed both by flow cytometry and the presence of chemokine receptors CXCR3 vs. CCR4) was increased in HD patients suffering from pruritus in comparison to those without this symptom. The authors reported that the concentration of inflammatory biomarkers was also increased (e.g., IL-6; reviewed in subsequent paragraphs). IL-6 is not the only cytokine which could play a role in CKD-aI. Previously, increased IL-2 concentrations have been reported among pruritic subjects with CKD [163]. IL-2 is a cytokine stimulating proliferation and generation of effector and memory T lymphocytes [164]. It exerted pruritic response after intradermal injection in healthy and AD subjects [165,166], whereas it was also more represented in the skin of patients with psoriasis suffering from pruritus [167]. Notably, IL-2 has been utilized therapeutically (e.g., in metastatic renal carcinoma or melanoma), with pruritus not infrequently occurring as an adverse event [168]. Fallahzadeh et al. [163] discussed the possible mechanisms linking IL-2 to CKD-aI and hypothesized that IL-2 may influence central nervous system (CNS), possibly by affecting physiologic functions of neural cells and neurotransmission or interfering with opioid receptors. Another physiological protein (IL-31) has been termed “pruritogenic cytokine” [169], although its effects in terms of inducing pruritus in healthy or atopic subjects are not immediate [170]. Unlike other members of the IL-6 family, it does not interact with gp130. The heterodimeric IL-31 receptor complex (IL-31R) consists of IL-31R*α* subunit and oncostatin M receptor β subunit (OSMRβ) [169]. IL-31 is produced mainly by Th_2_ lymphocytes and mature DCs [171] and exerts its biological effects through Janus-kinase (JAK1/JAK2) and other pathways (reviewed in details by Furue et al. [172]). The association between elevated IL-31 and CKD-aI has already been reported by several investigators [173,174], although the blockade of this pathway by nemolizumab (an anti-IL-31 monoclonal antibody) has not provided particularly effective outcomes in a recent phase II study (*n* = 69) [175]. Nevertheless, it seems that overexpressed IL-31 may contribute to CKD-aI through the activation of peripheral sensory nerves [174], as the expression of IL-31Rα was documented in a subpopulation of TRPV1+/TRPA1+ neurons of dorsal root ganglia in mice [171]. Another study on mice revealed that IL-31 promotes nerve elongation and branching in vitro and in vivo, which may play a role in pruritus associated with AD [176], possibly in other pruritic conditions with elevated IL-31 levels as well.

The pathogenetic considerations on CKD-aI are not comprehensive without covering the role of MCs. Classically, MCs were regarded as effectors of early and late phases of allergic reactions [177]. Allergic reactions in type I hypersensitivity are mediated by specific IgE antibodies which stimulate MCs through high-affinity IgE receptors (FCεRI). Subsequently, a plethora of mediators are released, resulting in neurogenic inflammation, which also includes wheals, flare, pruritus, oedema and sensitization of peripheral nerve endings [178]. The main pruritogenic mediators released from MCs are histamine, serotonin, tryptase, chymase, IL-31, leukotrienes, prostaglandins and neuropeptides [177,179]. Several studies correlated increased serum levels of histamine [180,181,182] and serotonin [183] with CKD-aI, whereas other studies were unable to demonstrate such associations [184,185,186]. Apart from “basic” IgE-related pathway leading to the release of histamine and serotonin from MCs (histaminergic pruritus), recently a new pathway has been discovered [187]. Non-histaminergic pruritus is elicited when MCs are stimulated by pro-adrenomedullin peptide 9–20 (PAMP 9–20) and compound 48/80, leading to abundant secretion of tryptase. The latter excites sensory nerves possessing receptors belonging to the family of Mas-Related G-Protein-Coupled Receptors (Mrgpr). The importance of tryptase in developing pruritus stems from its ability to cleave and activate PAR-2 receptor on sensory nerves, immune cells and even MCs themselves [179]. In fact, Dugas-Breit et al. [188] reported increased serum levels of tryptase in CKD patients, with CI intensity correlating significantly with tryptase concentration (*p* = 0.014). It is vital that MCs and nerves are in close contact with each other and may even be regarded as a common physiological unit [189]. Several investigators have confirmed the associations between MCs and CKD-aI [190,191,192,193,194], although German investigators reported no relation between the number of MCs present in the skin and the occurrence of pruritus [184]. According to different researchers, MCs were more prevalent in dermis of CKD-aI patients on HD [190,191,192], but also diffusely spread and highly degranulated [191]. Our group has previously demonstrated higher counts of tryptase- and chymase-positive MCs in HD patients than in healthy controls [193] and their susceptibility to apoptosis following UVB irradiation [194], with the latter findings constituting a rationale to manage CKD-aI with phototherapy.

### 2.3. Opioid Transmission

Opioid system is a physiological component of each organism, with a widespread distribution in both peripheral (PNS) and central nervous system (CNS) [195]. Feng et al. [196] reviewed diverse contributions of opioid system to the well-being of an organism, mainly its participation in ionic homeostasis, cell proliferation, neuroprotection, hibernation, pain modulation, emotional response, immune response, feeding, respiratory control and cardiovascular regulation. Essentially, opioid system is physiologically based on interactions between endogenous ligands and their receptors. The former include Met- and Leu-enkephalin, dynorphins (A and B), neoendorphin and β-endorphin. These ligands may interact with four major types of transmembrane G protein-coupled receptors: µ- (MOR), κ- (KOR), δ- (DOR) and nociception/orphanin (NOP-R) [197]. The physiological components may be interfered with in the setting of various diseases (e.g., addiction, depression, Alzheimer’s disease to name just a few [198,199,200]), or as a result of iatrogenic intake of opioids. Notably, exogenous opioids lead to numerous adverse effects, including pruritus, which affects 2–10% of patients following systemic administration [201]. The risk of pruritus is especially high if epidural, intraspinal or intrathecal route is chosen-with the latter, the reported prevalence may reach 100%. The influence of opioid system on the pathogenesis of pruritus is complex. Although certain opioids (e.g., morphine) can induce histamine release, others do not (e.g., fentanyl). Moreover, antihistamines are not effective in managing opioid-induced pruritus [202]. Experiments conducted on monkeys revealed that MOR agonists stimulated pruritic response, whereas MOR antagonists and KOR agonists attenuated pruritus [202,203,204]. In a recent study, Wang et al. [205] have reported that intrathecal administration of morphine in mice elicited scratching response due to activation of MOR receptors in spinal inhibitory interneurons, which resulted in disinhibition of spinal itch circuit. Studies in humans revealed decreased KOR expression in the skin of pruritic patients suffering from psoriasis vulgaris [206,207] and AD [208]. Accordingly, our group has been the first to demonstrate that HD patients suffering from CKD-aI (*n* = 21) had lower expression of KOR receptors than those without pruritus (*n* = 19; *p* < 0.02). Additionally, pruritus intensity was also inversely correlated with KOR expression (r= −0.63, *p* = 0.002) [209].

It must be noted that a study published already in 1995 assessed the concentration of Met-enkephalin in 21 CKD patients on HD [210]. When compared to healthy controls (*n* = 10), a statistically significant difference in Met-enkephalin concentration was noted (*p* < 0.01). However, the concentration of Met-enkephalin was not correlated with pruritus severity. No further studies on a larger sample of CKD patients have been executed. Interestingly, according to EUTOX database, Met-enkephalin and β-dynorphin are also listed as UTs from the MM group [45]. Given the mixed results concerning the association of β-dynorphin with pruritus in the course of various chronic inflammatory dermatoses [211,212,213,214], we have therefore decided to re-evaluate the possible role of several UTs, including endogenous opioids, in the context of CKD-aI in adults; as of June 2021, the study is in progress.

### 2.4. Infectious Agents

As mentioned previously, patients with CKD have a higher risk of infections. The impairment of adaptive T-cell responses may predispose to severe viral infections [215]. Based on epidemiological studies encompassing large groups of participants, infections with hepatitis B virus (HBV) and hepatitis C virus (HCV) are statistically related to the presence of CKD in the general population [216,217]. While chronic hepatitis may contribute to the development of CKD and its further progression to ESRD [218,219,220,221], patients may also acquire these infections *de novo*, especially if they are undergoing dialysis [222,223]. Among numerous complications associated with viral hepatitis, pruritus concerns 11–46% HBV patients and 22–58% of those with HCV [224,225,226,227]. Based on the data gathered from 18,801 patients on HD, it seems that concurrent HCV infection increases the risk of moderate to extreme pruritus (10% vs. 8.6%; *p* = 0.006) [22]. Abdelsalam et al. [228] have also demonstrated significantly higher pruritus severity among 193 HD patients with concomitant active HCV infection (*p* = 0.009). Interestingly, a Peruvian study has reported a decreased risk of severe pruritus with the coexistence of hepatitis C (*n* = 264 ESRD patients) [229]. As reviewed by Alhmada et al. [230], the mechanisms linking HCV to pruritus involve the occurrence cholestasis and induction of interferon-stimulated genes, whereas the main pruritic mediators include cytokines and chemokines (e.g., IL-8, CCL2, CXCL1, CXCL5). We were unable to find any studies which assessed pruritus prevalence and its severity among children suffering both from CKD and viral hepatitis. Due to the common practice of anti-HBV vaccination in developed countries and based on the studies on adults, the potential role of HCV seems more substantiated.

In 1985, Lempert et al. [231] used the term “pseudouremic pruritus” to elegantly illustrate a scabies outbreak in a dialysis unit in West Virginia, USA. Although we have never encountered similar situation in Poland, this report highlights that the presence of pruritus in a patient with CKD can also occur due to other causes. If scabies is not recognized and properly treated, intense and often generalized pruritus may bother the affected patient for weeks or months, fulfilling the criteria of CI, but wrongly attributed only to the underlying CKD or associated drug use. Notably, pruritus associated with scabies, as well as CKD-aI, frequently exacerbates at night. A recent onset of pruritus in a patient diagnosed with CKD may denote natural disease progression or an infestation with scabies, which is regarded as a “great mimicker”, occasionally tricking even experienced dermatologists. On the other hand, Yates et al. [232] have also reviewed the peculiar association between scabies and secondary streptococcal infection, which further predisposes to post-streptococcal glomerulonephritis (PSGN) and ultimately CKD.

### 2.5. Skin Dryness (Xerosis)

Xerosis is one of the most frequent dermatological symptoms which occurs in up to 29.4% of the general adult population [233]. It also constitutes a cardinal feature of atopic dermatitis, with the latter affecting at least 2–10% of young adults and 20% of children [234]. Therefore, due to other possible causes, the genuine prevalence of xerosis in children seems to be even higher. Xerosis concerns the majority of patients adult patients suffering from CKD (59.3–93.1%) [235], especially those undergoing dialysis. To the best of our knowledge, our research group was the first to assess the problem of xerosis among a cohort of CKD children (*n* = 102) [236]. In total, xerosis bothered 54.2% of patients, whereas in healthy controls it occurred less frequently (12.9%; *p* < 0.01). Regarding the basic CKD treatment approach, xerosis was present in 42.1% children on conservative treatment and 67.6% among those undergoing dialysis (*p* = 0.03). The control group presented with xerosis located only on the forearm and the lower legs. In CKD patients these were the most common locations, yet also more commonly involved than in controls; other locations were dry as well (the abdomen and the chest). Xerosis was also associated with the presence of pruritus (*p* < 0.01; detailed characteristics of pruritus in this cohort are reviewed in subsequent paragraphs). When comparing CKD patients with healthy controls, epidermal moisture evaluated by corneometry was lower on the lower legs, the abdomen and the chest (*p* < 0.001 each). Moreover, transepidermal water loss (TEWL) measured by tewameter was more pronounced in CKD than in healthy controls in certain locations, such as the forearms (*p* < 0.001) and the lower legs (*p* = 0.04). Notably, we noted correlations between xerosis and CKD-aI among children, confirming the results of previous studies conducted among adults [237,238,239].

The pathogenesis of uraemic xerosis seems multifactorial and the reported data is inconsistent when comparing different studies, especially in terms of xerosis-pruritus associations. Ståhle-Bäckdahl [240] evaluated 19 patients with CKD-aI undergoing HD and 12 healthy controls. There was no significant difference between stratum corneum (SC) water content between both groups. Park et al. [241] revealed decreased water content among patients on HD (*n* = 18) compared to healthy controls (*n* = 10) and decreased sweating response to sudorific agent, whereas there was no correlation between skin surface hydration and pruritus. Yosipovitch et al. [242] reported no influence of xerosis, SC hydration or sweat secretion on the occurrence of CKD-aI (*n* = 85). A Japanese study reported no correlation between pruritus intensity and SC water content by skin surface hydrometer, both before and after HD session [243]. We have previously evaluated skin hydration and lipid content in 80 ESRD patients on HD and 30 controls, demonstrating similar prevalence of xerosis in both groups (50% vs. 56.2%; *p* = 0.7) [244]. However, xerosis was more common in subjects complaining of pruritus (80% vs. 42%; *p* = 0.002). The presence of xerosis contributed to pruritus severity assessed by Visual Analogue Scale (VAS) among HD patients, whereas xerosis intensity demonstrated no such correlation. We have also demonstrated that SC lipid content differed between patients on HD and healthy controls. The former group presented higher content of ceramides 1, 2 and 3 (*p* < 0.01) and lower content of cholesterol and triglycerides (*p* < 0.01 each). Mixed results were also reported by Yosipovitch et al. [245], who observed impaired SC integrity in ESRD patients (*n* = 20) vs. healthy controls (*n* = 18; *p* = 0.001) and a correlation between decreased glycerol content and xerosis in the arm (*p* = 0.01). Other assessed characteristics of SC (permeability, pH and ultrastructure) did not differ between the groups. Despite such discrepancies in the pathogenesis, several clinical studies confirmed that emollient therapy ameliorates not only uraemic xerosis, but also CKD-aI [239,246,247].

### 2.6. Neuropathy

According to the statement “*it is the brain that itches, not the skin*”, the role of PNS and CNS in the generation of CI remains vital [248]. Essentially, CKD-aI is classified in the category of systemic causes of pruritus (III) according to IFSI classification, while “pure” neuropathic pruritus of central or peripheral origins is classified separately (V) [2]. However, certain publications reported that CKD may in fact contribute to neuropathy and possibly predispose to CKD-aI. Regardless, the reported findings seem inconsistent. Using neuron-specific enolase (NSE), Johansson et al. [249] demonstrated that nerve fibers and terminals were sprouting throughout the epidermis of 12 CKD patients on HD, in contrast to healthy controls (*n* = 15). However, there were no differences between patients suffering from pruritus and those free from this sensation. Fantini et al. [250] observed a reduction in the total number of nerve terminals in the skin when comparing 24 patients with CKD and 10 healthy subjects, although no correlations with pruritus were found. Zakrzewska-Pniewska et al. [251] reported that pruritus occurred among 32 of 51 ESRD patients on dialysis, with a higher severity of pruritus among individuals bothered by paraesthesia (*p* < 0.01). The signs of autonomic neuropathy did not correlate with pruritus. Papoiu et al. [252] conducted a functional magnetic resonance imaging (fMRI) of the brain in 13 ESRD patients on HD suffering from CI, revealing regional differences in grey matter density when compared to 15 healthy controls. Regional differences in brain perfusion were noted as well (detailed results therein).

To the best of our knowledge, there is a lack of data linking paediatric CKD, neuropathy and CI. Young patients with CKD may also suffer from peripheral neuropathy in up to 52%, although studies revealed a relative predominance of motor neuropathy over sensory [253,254]. Therefore, the hypothetic association with CKD-aI seems uncertain at most. In another study, multifocal white matter injury upon brain MRI scans concerned six out of twenty-nine children suffering from CKD (stages 4 and 5) [255]. Hartung et al. [256] reported several differences in grey and white matter volumes between young CKD patients (*n* = 85) and healthy controls (*n* = 63). Nevertheless, neurocognitive assessments demonstrated no differences in relation to regional brain volumes.

### 2.7. Dialysis Modality and Its Parameters

Along with the progression of CKD to ESRD, a patient is inevitably faced with a near prospect of dialysis or RTx. Various dialysis modalities may be considered as a crucial therapeutic intervention to decrease the bothersome signs and symptoms of CKD. However, regarding certain CKD manifestations, dialysis may also paradoxically contribute to their development and/or exacerbation. Accounting for the heterogeneity of available systems it is substantiated that the major differences regarding dialysis methods and parameters could exert influence on CKD-aI in a positive or negative direction. Historically, the initiation of HD in 1960s seems to have contributed to the increased prevalence of CKD-aI [257]. Early in 1970s the prevalence of CKD-aI reached 85%, further decreasing to 50–60% in the late 1980s [258]. Two decades ago our group evaluated 130 patients on HD, among whom 40.8% complained of CDK-aI [238]. There was a significant positive correlation between pruritus severity and duration of the HD period (*p* < 0.02). Patients undergoing dialysis on polysulphone membranes reported pruritus more commonly (57.9%) than on hemophane (30.8%; *p* < 0.04) or cuprophane (34.8%; *p* < 0.03) membranes. Aucella et al. [259] advocated the usage of polymethylmethacrylate (PMMA) membranes over standard low-flux synthetic membranes to diminish CKD-aI (*n* = 8). Chen et al. [75] demonstrated better effectiveness of high-permeability HD than conventional HD in terms of reducing CKD-aI intensity (*n* = 116). A randomized controlled trial (RCT) by Jiang et al. [260] revealed superiority of high-flux HD (HFHD) (*n* = 27) over HDF (*n* = 24) in reducing CKD-aI severity. At week 12, patients on HFHD experienced more significant reduction in mean VAS pruritus score (9.0 ± 1.8 points to 1.8 ± 0.4 points) than those on HDF (8.8 ± 1.6 points to 3.2 ± 0.8 points). Similarly, Ko et al. [261] reported that CKD-aI aggravation may be associated with low-flux HD (*n* = 111). Moreover, publications on dialysis patients consistently mention Kt/V value. This parameter was introduced over 30 years ago and refers to “dialyzer clearance of urea multiplied by dialysis time and normalized for urea distribution volume”, as reviewed by Vanholder et al. [262]. Kt/V value was also assessed in several studies focusing on CKD-aI, providing inconsistent results. For example, Zucker et al. [263] and Chen et al. [75] found no correlation between this parameter and CKD-aI occurrence and severity. Zucker et al. [263] emphasized that Kt/V value essentially denotes the clearance of LMWM, while the removal of MM and large molecules cannot be quantified this way. Therefore, even seemingly high Kt/V value does not necessarily prevent the accumulation of other UT which can theoretically predispose to CI. Still, the associations between Kt/V value and CKD-aI were in fact demonstrated in other papers [89,261,264]. One study reported the threshold Kt/V value of 1.5, with higher values favouring lower intensity of CKD-aI [261]. It is unknown if the observations on Kt/V values in adult patients with CKD-aI can be extrapolated to children with this symptom. Nevertheless, there is a plethora of evidence that the usage of Kt/V value as a dialysis adequacy marker must be carefully adjusted in a paediatric setting [265].

### 2.8. Other Factors

#### 2.8.1. Vitamin A

The rationale for investigating vitamin A in the context of CKD-aI stems from the observation that certain signs and symptoms of hypervitaminosis A (xerosis, pruritus) are also relatively common in CKD [266]. As reported by de Kroes and Smeenk [267], CKD patients had elevated serum vitamin A levels (*n* = 25). However, no correlation was found between vitamin A concentration and the presence of CKD-aI. Berne et al. [266] determined elevated retinol content in epidermis and serum of patients with CKD-aI (*n* = 10) when compared to controls (*n* = 5). After performing a whole-body phototherapy (UVA + UVB), pruritus disappeared in seven patients. Concurrently, epidermal content of retinol was reduced as well, whereas its serum concentration was not influenced by phototherapy.

#### 2.8.2. Aluminium (Al)

CKD patients may be subjected to increased exposure to aluminium (Al) due to different causes. Several decades ago, the main reasons included the contamination of the water used as a dialysate solution and the usage of phosphate binders [268]. Accumulation of Al predisposes to several complications of CKD, e.g., osteodystrophy, anaemia and encephalopathy [269]. Friga et al. [270] reported that elevated Al levels correlated both with CKD-aI occurrence (*p* = 0.008) and its intensity (*p* = 0.007) (*n* = 94). In a recent study (*n* = 866), Hsu et al. [268] revealed significant correlations between increased Al concentration in serum and CKD-aI. Moreover, each tenfold increase in serum Al concentration led to a 5.64 times higher risk of CKD-aI.

#### 2.8.3. Air Pollution

The magnitude of air pollution is constantly expanding in different regions of the world. Given the multifactorial nature of CI, it is worth exploring whether environmental factors actually contribute to its presence or intensity. Over the past decade, the associations between CI and air pollution have been explored in the context of children with AD [271,272,273]. As reported by Whang et al. [274], internet search queries for the word “itch” in fact correlated with the concurrent concentration of atmospheric particulate matter <2.5 μm (PM 2.5). Interestingly, chronic exposure to air pollutants have already been linked to the presence of CKD-aI in Taiwanese patients on HD (*n* = 866). The mean concentrations of nitric dioxide (NO_2_) and carbon monoxide (CO) during the previous 12 months correlated with the presence of CKD-aI (*p* < 0.001; *p* = 0.007; respectively) [275]. Additionally, increased concentrations of PM 2.5 for more than 116 days during the previous 12 months were also associated with CKD-aI (OR 3.57; *p* < 0.001) [276]. The authors summarized the possible pathogenetic mechanisms of their findings, including the stimulation of oxidative stress, skin barrier disruption, increased number of epidermal nerve fibers, immune dysfunction and neurogenic inflammation [275,276].

#### 2.8.4. Protective Role of Loop Diuretics

In 2017, Hayani et al. [277] reported that the intake of loop diuretics (furosemide or torasemide) during previous 12 months was associated with a significantly lower risk of pruritus among over 800 adult CKD patients on HD (25% vs. 15%; *p* < 0.001). Logistic regression analysis revealed adjusted odds ratio (aOR) of 0.622 for the current presence of pruritus (95% confidence interval 0.412–0.938; *p* = 0.024). The authors suggested several possible explanations for these observations, such as preservation of residual kidney function (RKF), anti-inflammatory effects of diuretics and binding to NKCCK ion channels. Cautious interpretation is warranted, especially in terms of potential direct causal relationship between loop diuretics and CKD-aI. Nevertheless, we deem these unexpected findings especially interesting as furosemide is one of the few drugs which are both officially registered for use in paediatric population and well-studied in clinical practice.

## 3. Epidemiology and Clinical Picture of CKD-aI in Children

Based on our literature search, Silverberg et al. [39] were the first authors to report on the subject of CKD-aI in children. Their study focused on cutaneous manifestations in 30 children of colour who suffered from chronic renal failure. The analysed group consisted of 10 children after RTx, 16 on dialysis and four managed with pharmacotherapy. All patients presented with skin conditions, especially xerosis, which bothered 86.7%. Pruritus was the second most common complaint, affecting 17 patients (56.7%). Regarding the subgroups, pruritus was reported by 25% in the conservative treatment, 69% in the dialysis and 50% in the transplant group. As stated by the authors, “*xerosis was often accompanied by pruritus*”, although a small number of participants precludes drawing definite conclusions. In 2002, Mettang et al. [40] published an editorial comment on CKD-aI. Therein, they recounted German data on 199 children and 505 adults on HD. CDK-aI was present in 9.1% and 18.8% patients, respectively (*p* = 0.0013). It was mentioned that “*the intensity was not very severe in the affected patients*”, although no further details were provided. The authors suggested these differences may be explained by a higher Th_1_ lymphocyte differentiation occurring in the course of aging. Subsequently, Turkish investigators [42] reported on 27 paediatric patients (aged 5–18 years) undergoing PD for at least 6 months. CKD-aI bothered 6 children (22.2%) among whom four were girls. Pruritus intensity according to VAS was very low (mean 1.33 points), ranging between 1–2 points. Gradual onset of pruritus was reported by four patients, whereas sudden by two. Pruritus was localized in five children; none of the pruritic patients provided any data on the factors aggravating the sensation, whereas all of them reported intermittent characteristics of pruritus. Interestingly, certain laboratory parameters were correlated with pruritus: serum phosphorus (*p* = 0.027), Ca × P (*p* = 0.031), PTH (*p* = 0.03) and CRP (*p* = 0.026). Another study on cutaneous disorders associated with uraemia included 43 children on HD and 38 healthy controls [43]. Xerosis was the most common symptom (53.5%), followed by pallor and pruritus (18.6% each). However, there was no difference in pruritus prevalence between uraemic subjects and controls (*p* = 0.606).

In 2016, our group published results on pruritus among Polish children suffering from CKD (stages 3–5) [44]. The study group consisted of 34 subjects on dialysis (20 HD, 14 PD), 38 subjects on conservative treatment and 31 controls. Overall, CKD-aI concerned 20.8% CKD patients (*n* = 15), with the mean pruritus intensity (assessed by VAS; patients were asked to determine maximal intensity within previous 24 h) of approximately 3.5 points. The chosen method of treatment did not influence pruritus severity, duration or location. Notably, generalized pruritus was more common in patients on dialysis than on conservative treatment (87.5 vs. 28.6%; *p* = 0.02). Children with CKD-aI had significantly lower eGFR (median 13.2 vs. 21.0 mL/min; *p* = 0.03), higher Ca × P (*p* = 0.015) and more frequently exhibited xerosis (66.7 vs. 50.9%; *p* < 0.01). Among the group of patients with CKD, those suffering from pruritus were also more prone to develop lower leg xerosis (60.0% vs. 21.1%; *p* < 0.01). We emphasize the difficulties in conducting studies in children with CI. Due to potential heterogeneity of age, especially the youngest respondents may not adequately express their sensations on itch intensity. A fraction of the data may therefore be obtained from parents/caregivers, or, based on scratching activity, assessed indirectly during the interview or sleep. Additionally, scratching leads to secondary skin lesions, e.g., excoriations, erosions, crusts, and skin infections. More chronic scratching leads to hypo- and hyperpigmentation, scarring and PN. We were unable to find any papers on the latter in children suffering from CKD; we speculate that CKD-aI intensity in paediatric population is generally too low to result in such outcome.

## 4. Management of CKD-aI in Children

Given the paucity of reports focusing on CKD-aI in paediatric population, its treatment may be regarded, without any overstatement, as *terra incognita*. Still, in subsequent paragraphs we have provided recommendations on the management of paediatric CKD-aI based on the available literature evidence on CKD-aI treatment in adults as well as CI treatment in children in general (Table 3). Taking into consideration the growing knowledge on UTs (especially PBUT) and strategies attempting to minimize their detrimental effects in CKD patients, we have also reviewed these aspects in terms of possible alleviation of CKD-aI in children. However, based on the current IFSI guidelines [1] it is vital to inform the parents/caregivers of a child suffering from CI about basic recommendations which are universal and in fact constitute the first therapeutic step. These include soft and permeable clothing (e.g., cotton), usage of non-alkaline soaps or syndets (synthetic detergents), short bathing in luke-warm water and avoidance of vigorous skin rubbing with towels after bath. Concurrently, patients should avoid any activity which predisposes to xerosis, such as too frequent and too long bathing, dry climate or alcohol compresses. Dietary aspects should also be discussed, with the intake of hot and spicy foods, hot drinks or alcohol firmly discouraged. Psychological factors such as excitement, strain or negative stress can also exacerbate CI. Therefore, relaxation techniques and education are also of benefit. To enhance patient’s compliance, all recommendations should also be provided in a written manner.

### 4.1. Topical Therapy

Regardless of the underlying aetiology of CI, topical therapy remains the mainstay of the treatment due to its safety and versatility. Moreover, taking into account the role of xerosis in developing CKD-aI, we deem emollients the crucial therapeutic step in managing children with this particular condition. The application of emollients results in a thin layer of occlusive lipids which limits TEWL [278]. Due to various ingredients, they replenish skin barrier, decrease inflammation and alleviate CI. The main antipruritic substances present in emollients include urea (5–10%), glycerol (20%), camphor (2%), menthol (1%), zinc (10%), pramoxine (1%) or polidocanol (2–10%) [1]. Although the cost or the regional unavailability of certain ingredients can be a major obstacle in their preparation, the pharmaceutical composition makes emollients an individually tailored modality, suitable for the needs of each patient. Emollients should be applied frequently (several times a day) and in high quantities. Their application is particularly useful shortly after finishing bath when skin is still moist. Notably, emollients have already proven beneficial in managing CKD-aI in adults. The most relevant studies in this topic assessed 15% glycerol and 10% paraffin emulsion [239], 10% urea and dexapanthenol lotion [279] and 2.2% gamma-linolenic acid cream [280]. Additionally, the use of topical anaesthetics in CKD-aI is also supported by clinical studies, e.g., concerning 1% pramoxine lotion [281] and bath oil containing polidocanol [282]. Our group has also successfully assessed the preparation containing structured natural lipids and endocannabinoids (N-acetylethanolamine and N-palmitoylethanolamine) in managing CKD-aI [247]. Apart from “pure” moisturizing effect, the mechanism of action may involve interference with histamine release or the activation of MCs.

Capsaicin is also mentioned among topical preparations alleviating CKD-aI [283,284,285]. This chilli-derived substance exerts its antipruritic properties via depleting substance P (SP) from sensory nerve terminals of the skin [286]. Due to skin-irritating properties, capsaicin preparations should be used cautiously in children. The use of topical calcineurin inhibitors (TCI) in CKD-aI gave mixed results. Two small studies reported improvement after 0.03% [287] and 0.1% [288] tacrolimus ointment, whereas a RCT reported no benefits of 0.1% ointment over placebo [289]. Moreover, 1% pimecrolimus cream was also ineffective in a RCT [290].

### 4.2. Phototherapy

Phototherapy with either UVA (320–400 nm; mainly as psoralen + UVA [PUVA] therapy) or UVB (290–320 nm) sources constitutes the second step in therapeutic ladder of several chronic inflammatory dermatoses, especially in psoriasis vulgaris and AD [234,291]. The fundamentals accounting for the effectiveness of phototherapy in dermatology are profuse and reflect close associations between the skin and the immune system. Essentially, phototherapy induces cytokine profile changes, apoptosis, immunosuppression and cell cycle arrest [292]. Following narrow-band UVB (NB-UVB; 311 nm) irradiation a threefold increase of p53 positive keratinocytes was noted, along with twelvefold increase of apoptotic cells and twofold decrease of Langerhans cells [293]. The role of broad-band UVB (BB-UVB) and NB-UVB in stimulating apoptosis of MCs has already been mentioned in previous paragraphs [194]. Other relevant mechanisms of phototherapy involve improved vitamin D status [294], reduced microbial colonization [295], increased production of antimicrobial peptides (AMP), enhancement of skin barrier [296] and influence on cutaneous sensory nerves [297].

Clinical studies on the efficacy of phototherapy in CKD-aI have been conducted since late 1970s. The majority of studies reported favourable outcomes after BB-UVB [298,299,300,301,302,303]. More recently, NB-UVB has been proven effective in several papers on CDK-aI [304,305,306,307], although one case report documented its lack of efficacy (and a better response to BB-UVB) [303]. Notably, Ko et al. [308] conducted a RCT comparing NB-UVB (*n* = 11) and placebo (UVA; *n* = 10), revealing no significant differences regarding pruritus intensity between both groups. Among secondary outcomes, NB-UVB provided significant improvement only in the body surface area affected by itch (*p* = 0.006). In a recent Cochrane systematic review on the management of CKD-aI the authors stated that “*UVB radiation may make little or no difference to uraemic itch (…) compared to UV-A/placebo*” [309]. Based on our experiences with NB-UVB in adult CKD-aI patients and in children with various inflammatory dermatoses, we conclude that this modality can be cautiously used in children older than 4 years with CKD-aI not responding to topical therapy.

### 4.3. Systemic Therapy

The instigation of systemic antipruritic therapy in children with CKD-aI or other types of CI is frequently hindered due to the lack of registration in these indications. Therefore, before starting therapy its off-label nature should be explained to the parents/caregivers of the patient. Potential nephrotoxicity of systemic drugs necessitates strict cooperation with an experienced paediatric nephrologist to prevent further progression of CKD to ESRD and to reduce the risk of other serious adverse events. In patients on dialysis, the dosing regimen should also be adjusted to its schedule, e.g., thrice a week.

#### 4.3.1. H_1_-Antihistamines

Among systemic drugs diminishing CI, H_1_-antihistamines have been used for decades. They are inverse agonists of H_1_-receptors, preferably binding to the inactive state of the receptor and stabilizing it [310]. First generation of H_1_-antihistamines encompasses, among others, hydroxyzine, clemastin, doxepin, dimetindene and ketotifen. These drugs are well-studied in children, although their antipruritic properties are usually small to moderate. The main benefit of first-generation H_1_-antihistamines includes their ability to cross blood-brain barrier (BBB) [311]. Although it results in decreased concentration and somnolence, the latter may be desirable in the evening or at night to improve sleep. An experimental study by Kim [312] reported that first-generation H_1_-antihistamines exert their effects on various subcortical and cortical regions of the brain which results in decreased pleasure associated with scratching. Regarding CKD-aI treatment in adults, the usage of hydroxyzine [313,314], doxepin [315,316] and ketotifen [185,317] is supported by the available literature. Concerning second generation of H_1_-antihistamines, desloratadine diminished CKD-aI in a prospective, crossover, open-label trial (*n* = 22). Unexpectedly, desloratadine turned out more effective than gabapentin in reducing pruritus intensity according to VAS (*p* = 0.004; *p* = 0.07; respectively) [318]. Second-generation H_1_-antihistamines are hydrophilic, do not cross BBB and are well-tolerated. However, setting aside the treatment of urticaria, their antipruritic properties are generally less prominent. Therefore, they may serve as active comparators to other investigated drugs. As an example, cetirizine failed to diminish CKD-aI in two studies, in contrast to naltrexone [319] and sodium thiosulfate [320].

#### 4.3.2. Gabapentinoids

The group of gabapentinoids encompasses two drugs which possess chemical structure derived from a physiological neurotransmitter, *γ*-aminobutyric acid (GABA). However, gabapentin and pregabalin in fact bind to *α*2*δ* subunit of presynaptic voltage-gated calcium channels (Ca_v_). This action hinders Ca^2+^ influx and prevents the release of excitatory neurotransmitters [321]. Gabapentinoids are mainly considered as antiepileptic drugs. Notably, they have also proven useful in the management of psychiatric disorders (such as bipolar disorder) and neuropathic pain [322,323]. Up to date, gabapentin [314,316,317,324,325,326,327,328,329,330,331,332] and pregabalin [315,327,328,332,333,334,335,336] constitute the most effective systemic treatment of CKD-aI in adults, with several hundred reported patients in the literature. Direct comparison between both drugs revealed no difference between their effectiveness [327,328,332], although gabapentin may be switched to pregabalin in case of insufficient tolerance. As mentioned in Cochrane systematic review on CKD-aI, GABA analogues have been studied in the highest number of RCT and both demonstrated the greatest effect size versus their comparators [309]. Importantly, the authors also emphasized that gabapentin is rarely mentioned as the first line of therapy in different guidelines on CKD-aI, while the most common practice still involves H_1_-antihistamines. Although we were unable to identify any literature on gabapentinoids and their usage in paediatric CKD-aI, their clinical value has been reported in children and adolescents suffering from post-burn pruritus [337,338,339,340].

#### 4.3.3. Ondansetron

Ondansetron is a competitive antagonist of serotonin 5-HT_3_ receptor and is used as a prophylactic and therapeutic agent against nausea and vomiting associated with chemotherapy, radiotherapy and surgery [341,342]. This drug was also used off-label (although with mixed results) in the treatment of pruritus associated with cholestasis [35,343,344,345,346,347] and prophylaxis of opioid-induced pruritus [348,349,350]. Despite the promising findings reported by Balaskas et al. [183], most of the later studies were unable to confirm the benefits of ondansetron in managing CKD-aI [335,351,352,353]. Nevertheless, based on our literature search, a case report by Deshpande [41] is the only paper which specifically mentions therapeutic aspect of CKD-aI in a child. Therein, the author recounts a case of a 15-year-old girl with systemic symptoms of uraemia and eGFR 10 mL/min. She was also bothered by severe pruritus which had started 8 months earlier. After starting PD, her clinical symptoms and laboratory parameters improved. However, pruritus worsened over the course of several months, appearing throughout the day and affecting sleep. The introduction of ondansetron (16 mg daily) resulted in a dramatic improvement and pruritus subsided within 2 weeks. Following withdrawal of ondansetron, pruritus promptly reappeared, with a subsequent disappearance due to reintroduction of the drug (8 mg/d). This case illustrates beneficial outcome of ondansetron in terms of CDK-aI resolution in a child, yet further studies on a larger group of young patients with this symptom are necessary to reach more definite conclusions.

### 4.4. Modalities Targeting PBUT

#### 4.4.1. Reducing Production

Reduced production of PBUT may be achieved by targeting early phases of the process. Several studies revealed that particular dietary interventions can decrease PBUT generation, including IS and pCS. The available literature supports the introduction of vegetarian diet [354], very low protein diet [355,356], Mediterranean diet [356] and higher intake of fibre [56,357,358,359]. However, protein restriction is not recommended in children with CKD as it has not been shown to influence the decrease in kidney function in children and may impair growth [360]. The findings by Rossi et al. [358] support the use of protein/fibre ratio due to better correlation with serum concentrations of both IS and pCS. Notably, El-Amouri et al. [56] documented an association between increased consumption of fibre and lower serum concentrations of free IS and free pCS in children with CKD. Other studies in humans demonstrated favourable effects on serum PBUT with the usage of probiotics [361,362], prebiotics [363,364,365] and synbiotics (probiotics and prebiotics used concurrently) [366,367,368,369,370]. In short, probiotics contain living species of certain beneficial bacterial strains which alter intestinal microbiota and decrease inflammatory state, whereas prebiotics are non-digestible substances present in food, stimulating growth and activity of certain bacteria in colon [115]. The introduction of both modalities may therefore result in lower production of UTs, especially PBUT. Nevertheless, some publications revealed no effect of probiotics on the generation of PBUT [371], including a Korean study conducted in children on HD [372].

In order to minimize the production of PBUT, other concept was introduced into practice as well. Essentially, constipation is a frequent symptom associated with CKD, affecting between 14.2–71.7% of dialysis patients [373]. As reported in the literature, accumulation of certain PBUT (especially pCS) may predispose to decreased bowel movements [374,375]. It is possible that pCS exerts proinflammatory effects which compromise bowel movements. On the other hand, if a constipation already occurs in a CKD patient, this could also predispose to a shift in bacterial metabolism, favouring proteolytic over saccharolytic activity [373]. Consequently, bacteria would be more prone to generate additional amounts of UTs and further perpetuate the process. These bilateral associations between impaired bowel mobility and the generation of UTs may have some practical implications, e.g., increasing dietary fibre intake to enhance bowel movements or to use laxatives. We underscore that there is no data which links such interventions in patients with CKD to the actual outcome of CKD-aI alleviation or disappearance, let alone in the paediatric population.

#### 4.4.2. Reducing Absorption

The overproduction of gut-derived UTs seems inevitable in advanced stages of CKD. Still, among many harmful substances, bacteria essentially produce the precursors of IS and pCS. This provides physicians with an opportunity to bind indole and *p*-cresole in the gut to minimize their absorption into the bloodstream, e.g., with activated charcoal. In fact, two studies have proven its effectiveness in managing adults with CKD-aI [376,377] (*n* = 34 in total), although none assessed if serum concentrations of IS, pCS or other UTs were initially elevated or concurrently diminished along with the alleviation of pruritus. Regardless, we deem these finding interesting due to several reasons. Firstly, activated charcoal is a safe treatment method with no serious adverse effects, although frequently associated with gastrointestinal symptoms. Interestingly, both studies on CKD-aI reported good tolerance [376,377]. As pointed out by Fusaro [378], it is also important to consider the use of other concomitant drugs as their absorption may be affected. Secondly, activated charcoal can be used even in small children if needed. Lastly, compared to many other drugs (including topical dermatologic preparations), it is relatively cheap, enhancing its availability to the patients in difficult economic situation. It seems that further studies on activated charcoal in the treatment of CKD-aI are required, with the particular emphasis on children.

AST-120 is an oral spherical carbon adsorbent, insoluble in water, and characterized by porous carbon particles [379]. Up to date, this compound has been approved in several Asian countries in the treatment of CKD. Although two major RCT demonstrated no definitive benefits in terms of diminishing CKD progression or mortality [380,381], a 1991 report by Niwa et al. [382] revealed a statistically significant decrease of serum IS concentration when compared to placebo after introducing AST-120 in a small group of CKD patients on HD (*n* = 26). This cohort also included ten patients initially suffering from generalized CKD-aI. In total, nine patients experienced any decrease in pruritus intensity, with five of them-completely. Although pruritic subjects did not differ from healthy controls in terms of baseline serum IS concentrations, the therapy with AST-120 led to a significant decrease of IS concentration (*p* < 0.05). We found no other studies linking AST-120 to the treatment of CKD-aI in adults nor any data on its use in children with CKD.

#### 4.4.3. Increasing Clearance

As mentioned in previous paragraphs, there is some evidence linking MM and PBUT accumulation to CKD-aI. Setting aside their actual causative and quantitative influence on CKD-aI, these UTs are difficult to be cleared, regardless of the dialysis modality chosen by the physician. Studies published up to date consistently reported that PBUT constitute the most difficult fraction to be cleared, even with the use of convective methods [383,384,385,386]. Moreover, according to Krieter et al. [387], instantaneous removal of IS and pCS via low-flux HD, HFHD or HDF cannot prevent their reappearance in a longer term. Brettschneider et al. [388] used fractionated plasma separation and adsorption (FPAD), with marked reduction ratio (RR) for both IS and pCS. According to a recent systematic review, this technique provided the highest RR concerning IS and pCS (78.2% and 71%, respectively) among the in vivo studies published up to date [389]. A combination approach to the removal of PBUT may also be beneficial, such as employing divinylbenzenic resin HD together with the intake of synbiotic [390]. Clearly, the removal of PBUT is a continuously evolving topic, with new concepts, materials, devices and techniques reported in recent years. The notable examples include mixed matrix membranes [391], direct hemoperfusion with activated carbon [392], liposome-supported HD [393] or ibuprofen infusion during HD [394], to mention just a few. Although the data in paediatric population is scarce, a recent study has evaluated the outcomes of post-dilution HDF, low-flux HD and HFHD in terms of PBUT clearance in children. Unfortunately, no differences have been observed in PBUT clearance after 12 months, regardless of the chosen modality [53]. It must be noted that post-dilution HDF did in fact result in more successful clearance of *β*_2_-M (*p* < 0.01), which is a MM already reviewed in previous paragraphs. In conclusion, it seems that RKF is still a crucial parameter, even in ESRD patients, which provides significant (although not complete) clearance of PBUT from the organism [52,395,396,397]. Ultimately, if this mechanism fails as well, RTx remains the only option capable of introducing better control of PBUT concentrations [398,399] and preventing, at least partially, the plethora of possible associated complications. Following RTx, still no guarantee can be made on the disappearance of CKD-aI. Nevertheless, it bothers significantly lower fraction of adult patients than previously [26,27,28]. Based on our personal experience, children initially suffering from CKD-aI also benefit from RTx, with the majority of young recipients finally free from this symptom (unpublished data).

## 5. Conclusions

CKD-aI is an important symptom in adult CKD population, with significant epidemiology, clinical characteristics and outcomes. Its pathogenesis is particularly complex, although certain main domains are now clearly established. These include metabolic disequilibrium, immune dysfunction, altered opioid transmission, coexistence of infections, xerosis, neuropathy and various dialysis-associated factors. The “classic” dermatological approach to CKD-aI management is based on topical therapy, phototherapy and systemic therapy. These modalities are mainly represented by emollients, NB-UVB and psychoactive drugs (especially gabapentinoids), respectively. In short, these methods aim to improve xerosis and replenish skin barrier, reduce inflammation and interfere with the conduction and processing of neural impulses during itch pathway. Moreover, as the data on the role of UTs (and PBUT in particular) in various aspects of CKD is rapidly growing, we are also witnessing new concepts linking UTs to the development of CKD-aI. It seems evident that targeting UTs, although still problematic, will play an increasing role in the comprehensive approach to numerous systemic complications of CKD, including CKD-aI.

Concurrently, based on the scarce available data, CKD-aI in children occurs less commonly and with a more benign course than in adults. Certain differences in the epidemiology of underlying disorders contributing to CKD are evident when comparing individuals from different age groups. Consequently, it is also unknown to what extent the pathogenesis of paediatric CKD-aI differs from the adult population. Provided that the data on the role of UTs (and PBUT in particular) in eliciting CKD-aI is still insufficient among adults, the extrapolation of such associations to children should be even more cautious. We recommend that the basic therapeutic approach to a child with CKD-aI should rely mainly on the meticulous surveillance and therapy by an experienced paediatric nephrologist, supported by the general antipruritic recommendations and the application of emollients. Should these modalities fail to diminish pruritus, the instigation of phototherapy or systemic medications may be considered in rare cases, following dermatological consultation.

## Figures and Tables

**Table 1:** Major groups of pathogenetic factors contributing to the development of CKD-aI.

Uraemic toxins (UTs) Immune dysfunction Altered opioid transmission Infectious agents Skin dryness (xerosis) Neuropathy Dialysis modality and its parameters Others

**Table 2:** Examples of UT according to the EUTOX database [45]. Asterisk marks substances linked to CKD-aI based on the available literature.

Substance Groups	Major Examples of UT
Free water-soluble low molecular weight molecules (LMWM)	Uric acid (UA) *, asymmetric dimethylarginine (ADMA), malondialdehyde, neopterin, *N*-methyl-2-pyridone-5-carboxamide, N-methyl-4-pyridone-3-carboxamide
Middle molecules (MM)	*β*_2_-Microglobulin * (*β*_2_-M), parathormone * (PTH), interleukin-6 * (IL-6), leptin, cystatin C, met-enkephalin *, *β*-dynorphin *
Protein-bound uraemic toxins (PBUT)	Indoxyl sulfate * (IS), *p*-cresyl sulfate * (pCS), homocysteine, carboxymethyllysine, hippuric acid

**Table 3:** Treatment modalities of possible usage in children with CKD-aI.

Therapeutic Group	Examples of Treatment
Topical therapy	Emollients
Phototherapy	NB-UVB
Systemic therapy	H_1_-antihistamines, gabapentin, pregabalin, ondansetron, activated charcoal
Targeting PBUT	Reducing production: proper protein intake, fibre intake, probiotics, prebiotics, synbiotics, laxatives
	Decreasing intestinal absorption: activated charcoal, AST-120
	Increasing removal: preserving kidney function, improving dialysis, RTx

## Data Availability

Not applicable.
